# Notch and Prospero Repress Proliferation following Cyclin E Overexpression in the *Drosophila* Bristle Lineage

**DOI:** 10.1371/journal.pgen.1000594

**Published:** 2009-08-07

**Authors:** Françoise Simon, Pierre Fichelson, Michel Gho, Agnès Audibert

**Affiliations:** 1 Université Pierre et Marie Curie-Paris 6, UMR 7622, Paris, France; 2 CNRS, UMR 7622, Paris, France; National Institute of Diabetes and Digestive and Kidney Diseases, United States of America

## Abstract

Understanding the mechanisms that coordinate cell proliferation, cell cycle arrest, and cell differentiation is essential to address the problem of how “normal” versus pathological developmental processes take place. In the bristle lineage of the adult fly, we have tested the capacity of post-mitotic cells to re-enter the cell cycle in response to the overexpression of cyclin E. We show that only terminal cells in which the identity is independent of Notch pathway undergo extra divisions after CycE overexpression. Our analysis shows that the responsiveness of cells to forced proliferation depends on both Prospero, a fate determinant, and on the level of Notch pathway activity. Our results demonstrate that the terminal quiescent state and differentiation are regulated by two parallel mechanisms acting simultaneously on fate acquisition and cell cycle progression.

## Author Summary

Despite substantial progress that has been made, we still know little about how single precursor cells undergo a limited number of cell divisions before arrest. Discovering the mechanisms by which terminal cells maintain cell division arrest is essential for understanding “normal” development, as well as the origin of pathological deregulations. Using the bristle cell lineage, a model system widely employed to analye cell identity acquisition, we observed that only two out of four terminal cells in this lineage are unable to re-enter the cell cycle and proliferate. Our study shows that in these cells, cell division arrest is maintained by the action of the transcription factor Prospero and the signalling pathway Notch. Since both of these factors also control cell identity in this lineage, this finding demonstrates that common elements acting simultaneously and in parallel regulate the terminal quiescent state and differentiation. This system provides a unique animal model in which to understand how the mechanisms involved in cell fate acquisition and those controlling cell division intermingle to produce cell lineages resulting in terminal cells in the right number and at the right place and time.

## Introduction

A high degree of coordination between cell proliferation, cell cycle arrest and cell differentiation is essential for proper development. Disruption of this coupling can lead to malformations and eventually cancer. Cell cycle progression relies primarily on the activity of cyclin-dependant kinases (Cdk) that are regulated by their association with factors like cyclins or cyclin kinase inhibitors (CKI) and by phoshorylation or dephosphorylation [1]. In worms and vertebrates, this mechanism is redundant and inactivation of certain cell cycle factors can be compensated by the activation of others. This has been observed for Cyclin-E (CycE), which modulates Cdk2 activity and controls the transition from the G1 to S phase. In mouse and *C. elegans*, cell divisions are not completely blocked after genetic ablation of cycE [2],[3]. However, cycE^−/−^ mouse cells are resistant to oncogenic transformations suggesting that normal and oncogenic proliferation have different requirements for CycE [3]. In *Drosophila*, the core mechanism of the cell cycle is not redundant and down-regulation of CycE arrests the cell cycle. Thus, CycE appears to be the most important G1 cyclin in all *Drosophila* cell divisions studied so far. In addition, it has been shown that ectopic expression of CycE after terminal mitosis induces re-entry into the S-phase resulting in additional cell cycles [4]–[6]. This shows that terminal cells continue to respond to CycE, and suggests that, as in vertebrates, *Drosophila* CycE seems to be central to the generation of ectopic rounds of cell divisions after cell cycle deregulation.

**Figure 1:**
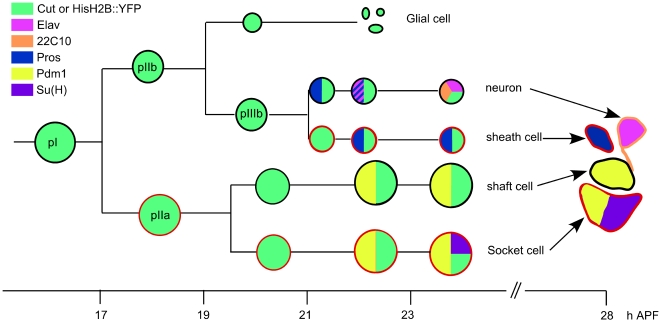
Schematic representation of the wild type bristle lineage and cell markers used. Cells are represented by circles showing the relative nuclear size with the expression of five molecular markers indicated in colour. On the right, a sensory organ conserving the relative position of each cell, anterior is on the top. Cells encircled in red are N-on cells and other the N-off cells. All cells are identified by Cut immunoreactivity or after *neur>HisH2B::YFP* overexpression (green). Markers: Prospero (Pros) in blue; ELAV in pink; 22C10/Futsch in orange; Pdm1 in yellow and Suppressor of Hairless (Su(H)) in purple. Time (h) APF is indicated at the bottom.

In the *Drosophila* bristle lineage, which produces the external mechanosensory organs called microchaetes, cell cycle progression and cell determination are intimately related [7]. Each adult microchaete is composed of four cells: two outer cells, the socket cell and the shaft cell, and two inner cells, the neuron and the sheath cell [8]. Each cell differs from the other by its size, localisation in the cluster and expression of specific markers (Figure 1). All four cells arise from a unique precursor cell, pI, after four asymmetric cell divisions occurring during early pupal development. At each division, one daughter cell (N-off) acts as a Notch ligand-producer and the other (N-on) as a Notch signal-receiver [9],[10]. The bias in the activation of the N-pathway assured the acquisition of different fates by both daughter cells. During the first round of division, the pI cell divides at about 16 h after puparium formation (APF) and generates two secondary precursor cells, pIIa and pIIb. During the second round of mitosis, the pIIb cell divides prior to the pIIa cell giving rise to a glial cell and a tertiary precursor cell pIIIb. The division of pIIa generates the socket and the shaft cell. Finally, the pIIIb cell divides to produce the neuron and the sheath cell [11]. Later in development, between 21 to 24 h APF, the glial cell undergoes apoptosis [12]. Thus, only four cells of the bristle lineage form each sensory organ. Upon completion of the lineage, cells stop proliferating and terminally differentiate. This stereotyped lineage has become an excellent model to analyse the relationship between cell cycle and cell determination. In particular, to address questions as to how cells maintain their terminal quiescent state or whether all terminal cells are responsive to proliferative signals. To analyse these issues, we tested the capacity of post-mitotic cells to re-enter the cell cycle in response to the overexpression of CycE that mimicked the cell's response to a proliferative condition. Surprisingly, not all cells in the lineage are sensitive to this overexpression and we show that the responsiveness to ectopic proliferation depends on both Prospero (Pros), a transcription factor, and the level of Notch (N) pathway activity.

## Results

### Ectopic CycE overexpression leads to the formation of sensory organs with extra-terminal cells

**Figure 2:**
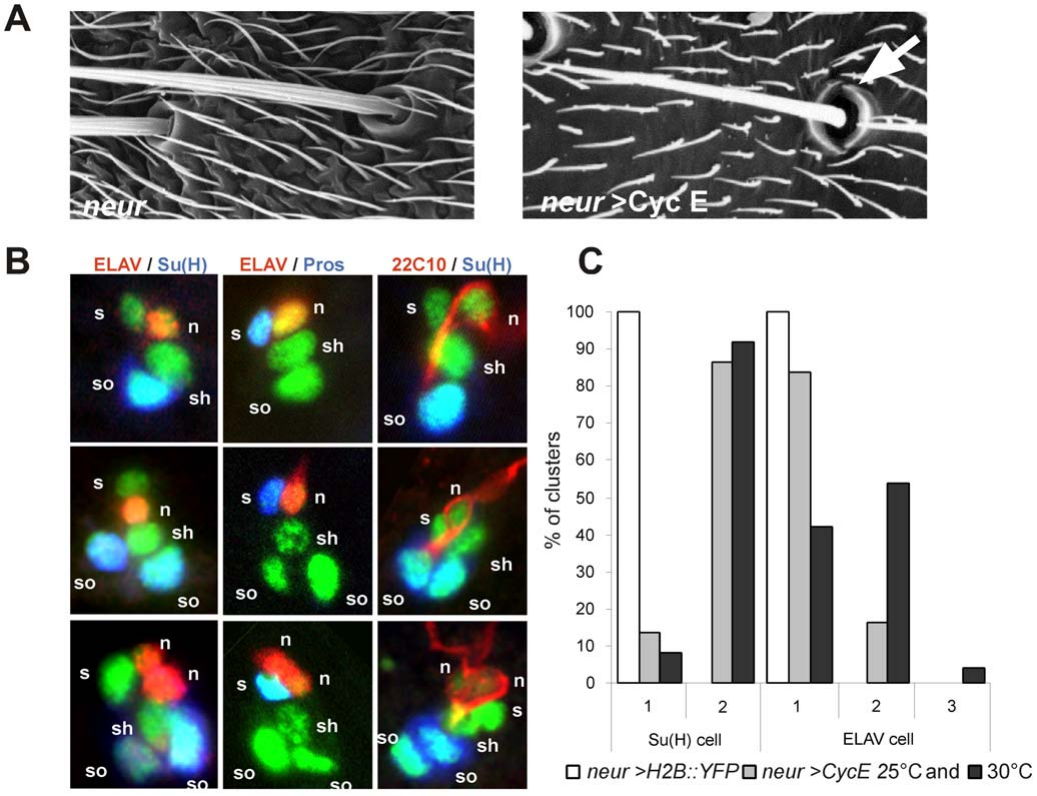
Extra socket cells and neurons are observed when CycE is overexpressed in the bristle lineage. (A) Scanning electron micrograph showing mechanosensory bristles of *neur* (left) and *neur>CycE* (right) flies. The arrow indicates duplicated socket cells observed after CycE overexpression. (B) Different types of sensory organs in *neur>CycE*, *H2B::YFP* pupae at 28 h APF. Sensory cells are in green (GFP staining). Socket cells are detected by their specific accumulation of Su(H) (blue in left and right panels), the sheath cells by Pros immunoreactivity (blue in middle panels) and neurons by ELAV (red in left and middle panels) and 22C10 immunoreactivity (red in right panels). n: neuron; s: sheath cell; sh: shaft cell; so: socket cell. (C) Quantification of the phenotype obtained after CycE overexpression. Histogram shows the percentage of clusters harbouring one or two socket cells and one, two or three neurons in wild type (white bars) or *neur>CycE* at 25°C (grey bars) and 30°C (black bars).

In order to analyse the mechanisms that maintain arrest in terminal cells of the bristle lineage, we forced proliferation by specific overexpression of CycE using the UAS/Gal4 system [13]. The *neuralized ^p72^*-GAL4 line (*neur*) was used as a driver to simultaneously overexpress CycE and Histone2B::YFP (H2B::YFP), which highlights the DNA [14]. After CycE overexpression, 84% of the organs contained two sockets as revealed by scanning electron microscopy (Figure 2A). At the cellular level, each sensory organ cell was identified by the expression of H2B::YFP (see Materials and Methods), socket cells by the accumulation of high levels of Suppressor of Hairless (Su(H)), sheath cells by the presence of Prospero, and neurons by the presence of ELAV or 22C10/Futsch (see Figure 1) [9],[11],[12],[15]. At 28 h APF, we observed three types of clusters. 13,5% of the clusters were wild-type and formed by four cells (Figure 2B, upper row, n = 22, and Figure 2C), 70% of the clusters contained one additional socket cell (Figure 2B, middle row, n = 115, and Figure 2C), and 16,5% of the clusters exhibited two additional cells namely, an extra-socket cell and an extra-neuron (Figure 2B, bottom row, n = 28, and Figure 2C). We never observed clusters with more than one shaft and one sheath cell. When overexpression of CycE was carried out at 30°C, where the GAL4/UAS system is more efficient, cluster composition was similar to that of pupae maintained at 25°C (Figure 2C, 92% of the clusters showed duplicated socket cells, n = 71, and 58% showed multiple neurons, n = 131). Similar results were obtained when cluster composition was analysed up to 48 h APF showing that after a time-lapse longer than six cell-cycles neither extradivisions nor apoptosis occurred (Figure S1 and data not shown). These data indicate that the duplication of neurons and socket cells are reproducible events induced after CycE overexpression and suggest that bristle lineage cells have differential sensitivities to proliferating signals.

### CycE overexpression induced extra mitoses in a cell type–specific fashion

To identify the origin of these additional differentiated cells, we carried out *in vivo* imaging to follow the formation of the bristle lineage in *neur>H2B::YFP*, *PON::GFP* (see supplementary data in [16] and Video S1) and in *neur>CycE*, *H2B::YFP*, *PON::GFP* pupae (Video S2). We used the PON::GFP fusion protein to identify the normal set of sensory cells during the time-lapse recording. Precisely, PON::GFP was inherited by the pIIb cell, the glial cell, the shaft cell and by the neuron [17].

Overexpression of CycE did not affect the sequence or the asymmetry of the first four divisions. Thereafter, up to two supplementary divisions were observed. One involved a pIIa daughter cell identified as the future shaft cell by its position in the cluster and its inheritance of Pon::GFP during pIIa mitosis (observed in 80% of clusters analysed, n = 124). The second involved a pIIIb daughter cell identified as the future neuron by its position and its inheritance of Pon::GFP during pIIIb mitosis (observed in 25% of clusters analysed, n = 124) (see Video S2 and Figure S2). This supplementary division was always observed in clusters where the pIIa daughter cell had also undergone an extra-division. Consistently, overexpression of CycE at 30°C led to 94,5% of the shaft cells and 45% of the neurons undergoing an extra-mitosis (n = 102).

**Figure 3:**
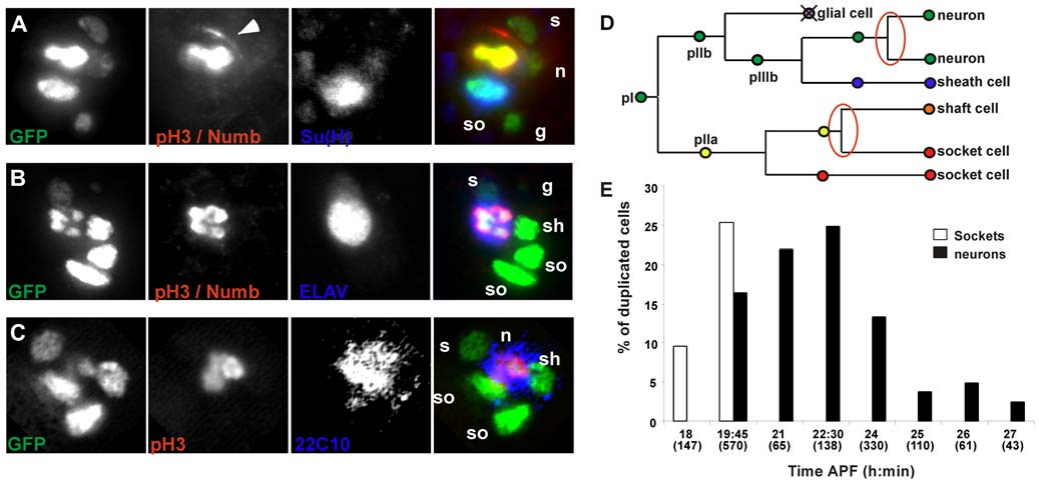
Extra mitoses occurred in the future shaft cells and neurons. (A–C) Mitoses are revealed by PH3 immunoreactivity (second columns and red in the super-imposed images, right panels); in the same panels, Numb immunoreactivity (arrowhead in the second column, red in the merge). PH3 and Numb are fully distinguishable by their respective nuclear and cortical locations. Socket cells and neurons are respectively identified using Su(H) and ELAV or 22C10 immunoreactivity (blue). Sensory cells are detected by their GFP immunoreactivity. (A) *neur>CycE*, *H2B::YFP* pupae at 21–22 h APF. An extra mitosis occurs in the future shaft cell, which is identified by nuclear size and lack of Su(H) accumulation. n: neuron; s: sheath cell; sh: shaft cell; so: socket cell; g: glial cell. (B) *neur>CycE*, *H2B::YFP* pupae at 24–25 h APF. Extra mitosis (red in the right panel) occurring in an ELAV positive cell (blue). (C) *neur>CycE*, *H2B::YFP* pupae at 24–25 h APF. Extra mitosis (red in the right panel) in differentiated neurons identified by the presence of the late neuronal-specific marker 22C10 (blue in the right panel). (D) Schematic representation of the bristle lineage after CycE overexpression. Extra mitoses are encircled in red. (E) Histogram showing the percentage of duplicated socket cells (white bars) and neurons (black bars) obtained after CycE overexpression induced by a heat shock applied between 18 h and 27 h APF.

To confirm the identity of the ectopically dividing cells, triple stainings were performed labelling metaphasic cells (phospho-ser10 histone H3 (PH3) immunoreactivity) together with bristle lineage cells, neurons or socket cells. Figure 3A shows a mitotic cell in a five-cell cluster from a pupae at around 22 h APF. This mitosis is the first additional division that we observed with time-lapse imaging. The mitotic cell was identified as the future shaft cell by its nuclear size, position and its lack of Su(H) accumulation. The second additional mitosis was observed in clusters from pupae at around 24 h APF. In this case, the mitotic cell corresponded to the neuron since it expressed ELAV (Figure 3B) and 22C10/futsch (Figure 3C) markers. The fact that in five-cell clusters an extra socket cell was observed suggests that the extra division of the future shaft cell gives rise to a shaft and a socket cell. Similarly, in later clusters, two neurons were observed suggesting that the extra-division of the future neuron gives rise to two neurons. These observations were confirmed in *in vivo* time-lapse followed by immunodetection experiments (not shown). Taken together these data indicate that the shaft cell and neuron are the only two cells in the terminal bristle lineage to undergo an ectopic division when CycE is overexpressed (Figure 3D).

### Neurons can divide late during differentiation

To further analyse cell behaviour in response to CycE overexpression, we sought to determine the critical period during which cells were competent to undergo additional mitosis. To do so, CycE was expressed under the control of a heat-shock promoter at different moments in the cell lineage. Antibody staining was carried out twelve hours after heat shock to distinguish socket cells and neurons. Figure 3E shows the percentage of clusters with duplicated socket cells or neurons following heat shock treatment at different times APF. Two socket cells were generated only when CycE was overexpressed at the time of pIIa mitosis (between 18 h and 19 h45 APF). In contrast, the period in which CycE overexpression was able to trigger a division of the neuron was longer (from 19 h45 to 26 h APF). Since axogenesis starts at about 23 h30 APF [12], ectopic neuronal divisions can occur at a time when neurons appear to be fully differentiated. This is shown in Figure 3C in which a dividing cell was positive for 22C10/Futsch, a late neuronal marker that reveals differentiated neurons [15]. Interestingly, in some cases we observed that the future neuron underwent two consecutive rounds of supplementary divisions (not shown). These data indicate that neurons retain their competence to divide late into differentiation.

### CycE overexpression induces an extra-asymmetric division in the pIIa sub-lineage

The data presented above indicate that the first extra mitosis is asymmetric and generates two different cells (a shaft and a socket cell) while the second one is symmetric giving rise to two identical cells (two neurons). In the normal bristle cell lineage all mitosis are asymmetric. The cell-fate determinants, Numb and Neuralized (Neu), co-segregate to one daughter cell, where both act to bias the Notch-mediated fate decision [18],[19]. We analysed the distribution of these factors in cells undergoing extra divisions following CycE overexpression. During the mitosis of the future shaft cell, Numb and Neu were detected and formed a crescent at the anterior pole of the mitotic cell (n = 14, Figure 3A, arrowhead and data not shown). The differential segregation of these factors was in agreement with the unequal repartition of the Pon::GFP fusion protein observed during *in vivo* recordings (see Video S2 and Figure S2). In contrast and in accordance with the symmetric characteristic of this division, Numb was never detected during the extra division of the future neuron (n = 9, Figure 3B). This is in agreement with *in vivo* recording data showing that Pon::GFP seems to be distributed uniformly between both daughter cells (Video S2). These data indicate that when cell divisions are forced in the pIIa sub-lineage, the future shaft cell behaves as its mother cell, pIIa, dividing asymmetrically and giving rise to another shaft and socket cell.

### Activation of the Notch pathway prevents extra cell mitoses in socket cells

**Figure 4:**
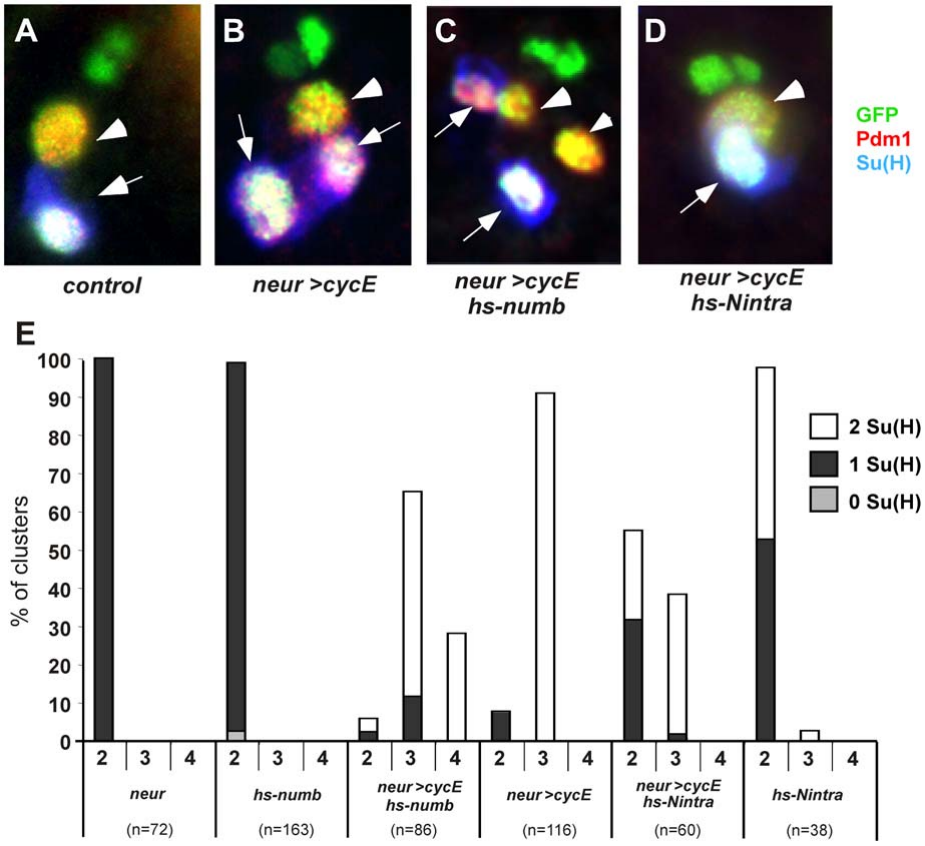
Activation of the Notch pathway prevents extra mitoses in the pIIa sublineage. (A–D) pIIa daughter cells are revealed by Pdm1 (red), socket cells by Su(H) (blue) and sensory cells by GFP immunoreactivity (green, UAS-H2B::YFP in all cases) in (A) control, (B) *neur>CycE*, (C) *hs-numb*, *neur>CycE*, and (D) *hs-Nintra*, *neur>CycE*. Pupae were heat shocked at 20 h APF and dissected after one night of recovery. Arrowheads indicate shaft cells and arrows socket cells. In (C), four pIIa daughter cells, amongst which two socket cells, are detected. (E) Histogram showing the percentage of clusters harbouring 2, 3, or 4 pIIa daughter cells in genotypes indicated on the abscissa. The percentage of clusters with no socket cell (grey bars) one socket cell (black bars), two socket cells (white bars) is indicated.

The extra division of the future shaft cell was not correlated to different levels of CycE overexpression (Figure S1). Moreover, both cells responded similarly to CycE overexpression, exhibiting precocious and synchronous entries into the S phase [20]. This suggests that certain factors favours the progression of the mitotic cell cycle in the shaft cell or alternatively, prevent division in the socket cell. As the N pathway is off in the shaft cell, its capacity to respond to CycE overexpression could be related to the absence of N pathway activation. To analyse this possibility, we controlled the activation of the N pathway concomitantly in both pIIa daughter cells with CycE overexpression (*neur>CycE*). N pathway was inhibited by expressing Numb under the control of a thermo-inducible promoter in both pIIa daughter cells (30 min pulse at 38°C at 20 h APF, [21]). Pdm1 was used as a marker of all pIIa progeny [16] and socket cells were identified by their accumulation of Su(H) (Figure 4A–4D). The percentage of clusters containing 2, 3 or 4 pIIa daughter cells obtained under different experimental conditions is shown in Figure 4E. In each case, the proportion of clusters harbouring 1 or 2 socket cells is indicated. Importantly, under these conditions Numb overexpression induced a very low rate of socket to shaft cell transformation (Figure 4E, hs-*numb* column 2). When CycE was overexpressed together with mild induction of Numb, we observed that 28% of the clusters harboured four pIIa daughter cells with two socket cells and two shaft cells (Figure 4C, E hs-*numb*, *neur>CycE* column 4). Under these same conditions, *in vivo* analysis showed that both pIIa daughter cells divided (not shown). Such clusters were never observed when CycE was overexpressed alone (Figure 4E, *neur>CycE* column 4). Thus, when N activity was reduced and CycE overexpressed, the future socket cell divided asymmetrically giving rise to a shaft and a socket cell. This suggests that, similarly to the shaft cell, the socket cell retains pIIa features shortly after birth. Taken together, these data show that full activation of the N pathway is necessary to prevent the future socket cell from entering mitosis upon CycE overexpression.

To further analyse the action of N on the proliferative capacity of terminal cells, we performed reciprocal experiments and activated the N pathway in the shaft cells. This pathway was activated after overexpression of the intracellular domain of the N receptor (N^intra^) [22]. The expression of N^intra^ was induced at 20 h APF by a 30 min pulse at 34°C. Under these conditions, only 45% of clusters were transformed (clusters harbouring two socket cells, Figure 4E, hs-*Nintra*, column 2). We took advantage of this mild penetrance to analyse the effect of CycE overexpression together with weak Notch dependant transformation. We observed that the number of clusters containing three pIIa daughter cells (two of which were socket cells) was reduced from 91% under CycE overexpression alone to 38% under conditions of N and CycE overexpression (Figure 4E compare *neur>CycE* column 3 and hs-Nintra, *neur>CycE* column 3). This reduction was associated with an increase in the number of clusters having a normal set of pIIa daughter cells (a socket and a shaft cells, Figure 4D and 4E, hs-Nintra, *neur>CycE* column 2). This indicates that the extra divisions induced by CycE overexpression were blocked by mild activation of the N pathway. These data show that in the pIIa sub-lineage, activation of the N pathway is involved in maintaining the future socket cell in a quiescent state. As a consequence, CycE overexpression induced cell division exclusively in the future shaft cell. A similar anti-mitotic action of the N pathway has been reported in *Drosophila* follicle cells. In these cells, lack of N activity has been shown to induce extra mitoses at the expense of endocycles [23],[24].

### Pros prevents extra cell mitosis in the pIIIb daughter cells

Similar to the situation in the pIIa sub-lineage, only the N-on cell of the pIIIb sub-lineage, namely the sheath cell, did not undergo extra mitosis upon CycE overexpression. To test whether activation of the N pathway was necessary to prevent ectopic division of the sheath cell, we reduced the activation of this pathway by overexpressing Numb (30 min heat shock at 38°C, at 21 h30 APF) together with CycE (*neur>CycE*). Under these conditions, the overexpression of Numb was not sufficient to modify cell identity, since both an ELAV and a Pros positive cell (neuron and sheath cell respectively) were present in all clusters. Surprisingly, even though the conditions of Numb overexpression were similar to previous experiments, we observed no change in the number of clusters with duplicated sheath cells when CycE was overexpressed (0% vs 4% in *neur>CycE* (n = 126) and in hs-*numb*, *neur>CycE* (n = 118) respectively). Similarly, no change was observed in the number of clusters with duplicated neurons (19% vs 22% in *neur>CycE* (n = 126) and in hs-*numb*, *neur>CycE* (n = 118) respectively).

**Figure 5:**
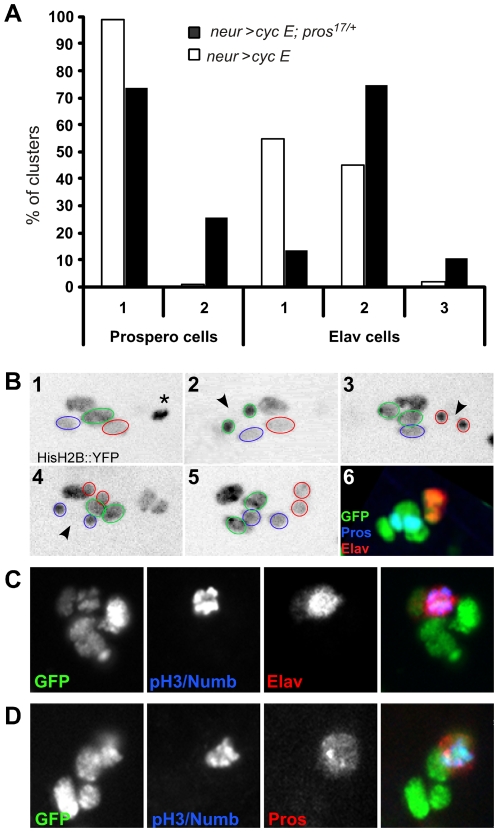
Pros prevents extra mitoses in the pIIIb sublineage. (A) Histogram showing the percentage of clusters containing one or two sheath cells and clusters with one, two or three neurons after CycE overexpression alone (*neur>CycE*) (white bars) and in a *pros17/+* background (black bars), at 28 h APF. (B) Combined time lapse imaging in living pupae and immunostaining of *pros17/+*, *neur>CycE*, *H2B::YFP*. Representative frames from the time-lapse recording are shown for one cluster. Cells encircled in green correspond to the future shaft cell and its progeny. Cells in red correspond to the neuron and its progeny and blue lines delimit the sheath cell and its progeny. The non-circled nucleus corresponds to the socket cell. (1) Cluster at 23 h APF showing the normal number of cells. The glial cell is undergoing apoptosis (asterisk). (2–4) Extra mitoses (future shaft in 2, neuron in 3 and sheath in 4) are indicated with an arrowhead. (5) Last frame of the movie. (6) Immunostaining of this cluster after the time-lapse recording. Neurons and sheath cells are identified by ELAV (red) and Pros (blue) immunoreactivity. Bristle lineage cells are revealed using an anti-GFP antibody (green). (C,D) No Numb protein was detected during pIIIb daughter cells division. The ELAV (C, in red) and Pros (D, in red) positive cells are mitotic cells as revealed by PH3 immunoreactivity (blue). *neur>CycE*, *H2B::YFP*, *pros17/+* pupae at 24–25 h APF.

The fact that the response of pIIIb daughter cells to CycE overexpression was invariant after Numb overexpression suggests that factors other than N can prevent mitosis in these cells. One candidate that may impede extra mitoses is the cell determinant Pros, as loss of function of *pros* results in ectopic mitotic activity in the *Drosophila* central nervous system (CNS) [25],[26]. In the bristle lineage, Pros is detected in the pIIIb cell during its division, is inherited by the neuron where it disappears rapidly and, at the same time, is expressed in the sheath cell (Figure 1) [11]. To analyse the putative anti-mitotic role of Pros in the bristle lineage, CycE was overexpressed in a *pros^17/+^* heterozygous background. Results depicted in Figure 5A show that 26% of the clusters contained duplicated sheath cells in *pros^17/+^*, *neur>CycE* (n = 129) compared to 1% in *neur>CycE* alone (n = 71). Interestingly, we also observed an increase in the percentage of clusters containing multiple neurons (86% n = 129). These results suggest that with CycE overexpression, Pros affects the proliferative properties of the sheath cell and the neuron even if in this later Pros is transiently expressed. Similar results were obtained by overexpressing CycE using a hs-*CycE* construct at the time of pIIIb division (22 h APF) in a *pros^17/+^* background (data not shown). To determine the origin of the duplicated cells, we combined time lapse imaging to follow the pattern of cell division and immunostaining to identify the fate of pIIIb daughter cells. Within the 25 lineages followed, three types of extra divisions were observed: (i) in 64% of the cases, only one pIIIb daughter cell underwent an ectopic mitosis, giving rise to two neurons; this is similar to what was observed upon overexpression of CycE alone (Figure 3); (ii) in 8% of the cases, three neurons were observed, the third one resulting from an extra cell division of a duplicated neuron (not shown); (iii) in 24% of the cases, both pIIIb daughter cells divided, giving rise to two neurons and two sheath cells respectively. An example of an *in vivo* recording of such a lineage is shown Figure 5B and Video S3. These data show that both pIIIb daughter cells underwent a symmetric division, giving rise to two neurons and two sheath cells respectively. The symmetric nature of these divisions was confirmed by the lack of Numb staining during these additional mitoses (Figure 5C) and Pros was equally distributed between both daughter cells (Figure 5D). These data indicate that in a *pros^17/+^* background, both pIIIb daughter cells are able to undergo an ectopic and symmetric mitosis in response to CycE overexpression. To determine whether loss of function of *pros* alone could also induce extra mitoses, we analysed the bristle lineage in *pros* null clones. Although CycE staining of pIIIb daughter cells inside *pros* clones was more intense, we failed to detect supplementary cell divisions (Figure S3). The absence of extra mitoses inside the *pros* null clones could be explained by the absence of Dacapo downregulation, Dacapo (Dap) being a CycE inhibitor.

Taken together, these data indicate that Pros has a dual function in the bristle lineage. In addition to its involvement in neuron and sheath fate determination [27], Pros acts as a cell cycle regulator in these two cells since it prevents extra cell divisions under conditions conducive to proliferation.

### Pros and Notch cooperate to prevent extra mitoses in pIIIb daughter cells

**Figure 6:**
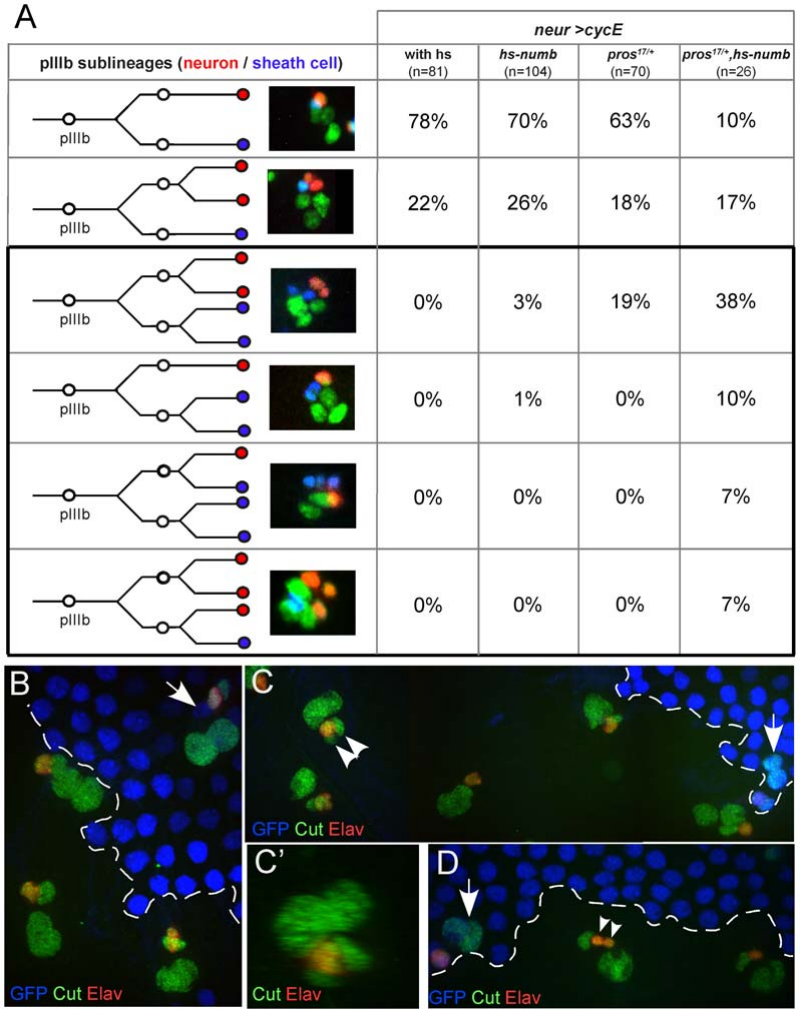
N and Pros cooperate to prevent extra mitoses in the sheath cell. (A) pIIIb sub-lineages were determined combining time lapse imaging and immunodetection in *neur>CycE* pupae alone or combined with *hs-numb*, *pros^17/+^* and *hs-numb*, *pros^17/+^*. Neurons and sheath cells were identified by ELAV (red) and Pros (blue) immunoreactivity. Cells of the bristle lineage were revealed using an anti-GFP (green) antibodies (UAS-H2B::YFP in all cases). The percentages indicate the proportion of each lineage in the different experimental conditions. The black box highlights the lineages where the sheath cell divides. The frequency of extra sheath cell division increased after combined reduction of Pros and N activity. (B–D) *pros^17^* clonal analysis in control (B) and *N^ts/+^* (C,D) backgrounds. Nota from pupae maintained at 30°C (restrictive temperature). Clones are detected by the lack of GFP staining (blue). Sensory organs and neurons are identified with cut (green) and ELAV (red) immunoreactivity respectively. (B) As in *pros* heterozygous tissue (arrows in B–D), sensory organs inside *pros^17^* null clones are formed by four cells (two big pIIa and two small pIIIb daughter cells). (C,D) in *N^ts/+^* background, several sensory organs inside *pros^17^* clones harbour three pIIIb daughter cells. Either one neuron and two sheath cells (arrowhead in C, and slightly tilted to reveal the two pIIa daughter cells in C'), or one sheath cell and two neurons (arrowheads in D).

The data presented above indicate that activation of the N pathway prevents mitosis of the socket cell upon CycE overexpression. A similar role is played by Pros in the sheath cell. Since the N pathway is active in the sheath cell, N and Pros could act redundantly to prevent extra divisions in this cell. To analyse this possibility, we overexpressed CycE (*neur>CycE*) while downregulating N activity (hs-*numb*) in a *pros^17/+^* background. Induction of Numb expression (30 min heat shock at 38°C) was performed under visual control on living pupae followed by immunostaining to identify sheath cells and neurons. Only clusters heat shocked within one hour following the pIIIb division were analysed. We observed a significant increase in the proportion of sheath cells that underwent an extra division (Figure 6A, black box): 62% in hs-*numb*, *pros^17/+^*, *neur*>CycE (n = 26) versus 19% in *pros^17/+^*, *neur*>CycE (n = 72) and 4% in hs-*numb*, *neur*>CycE (n = 104). These data indicate that decreasing N pathway activity in a *pros^17/+^* mutant background favours the division of the future sheath cell upon CycE overexpression.

The joint action of Notch and Pros on the maintenance of the quiescent state of pIIIb daughter cells has been revealed in a sensitized background of CycE overexpression. In order to analyse whether both N and Pros act similarly under normal conditions, we studied the composition of sensory clusters inside null *pros^17^* clones in conditions of down regulation of the N activity. This downregulation was obtained using pupae heterozygous for the thermosensitive allele of N, *N^ts-1^*, maintained at restrictive temperature of 30°C [28]. In control conditions (at a permissive temperature of 18°C) and using Cut immunoreactivity to identify lineage cells, we always observed that all sensory clusters inside *pros^17^* clones were composed by four cells, two pIIa and two pIIIb daughter cells, the former harbouring a nucleus bigger than the latter (Figure 6B, [12]). Similar results were observed under restrictive temperature (30°C) in heterozygous *N^ts-1^* pupae alone or in *pros^17^* heterozygous tissue (data not shown and arrows in Figure 6C–6D). However, in 8% of clusters inside null *pros^17^* clones, when N-function was reduced (at 30°C), we observed a supplementary pIIIb daughter cell identified by its small nucleus (arrowheads in Figure 6C–6D). In half of these cases, the supplementary cell was ELAV–positive indicating a neuronal identity. In the other half, the ELAV-negative cell probably corresponded to a sheath cell. These results suggest that ectopic cell divisions occurred when both Pros and Notch activities were reduced without forcing cell divisions by CycE overexpression. In the future sheath cell, the action of Pros appears predominant since reduction of the activity of the N pathway alone did not induce extra divisions after CycE overexpression.

## Discussion

In this study we have shown that in the bristle lineage terminal cells, the shaft cell and the neuron, but not the socket and sheath cells, undergo supplementary cell divisions after CycE overexpression. These supplementary cell divisions were not due to a cell-specific differential expression by *neur*>Gal4 of *cycE*. After CycE overexpression, CycE was detected in all cells of the cluster during the entire period analysed in *neur*>*cycE* pupae. This is in contrast with the absence of CycE detection in terminal cells under normal conditions [20]. However, between clusters, the level of CycE detected was variable in each cell type suggesting that the CycE accumulation was very dynamic (Figure S1). Nevertheless, we never observed a correlation between these variations and the cell-specific extradivisions reported. This absence of correlation was also found under the different genetic backgrounds used (Figure S1). In addition, we observed a similar cell-specificity using a heat-shock promoter construction to drive CycE. Taken together, these data show that the cell-specificity of the extra cell divisions observed was not due to an un-even CycE level driven by *neur*>Gal4. Furthermore, in previous studies, we have shown that all terminal cells are arrested in G1-phase [20]. This indicates that resistance to cycE overexpression was not due to a differential cell-cycle arrest in terminal cells of the sensory cluster. Finally, an ectopic S-phase was induced after CycE overexpresion even in cells that did not undergo extra divisions ([20], data not shown). This shows that the level of CycE driven by *neur*-Gal4 was sufficient to force cell-cycle progression in all cells. This suggests that inhibitory role of CKI, like Dacapo, was overridden by the accumulated levels of CycE. As such, these extra cell divisions are due to a *bone fide* behavioural difference of the shaft cell and the neuron. Indeed, we show that this difference is due to the action of the Notch pathway and/or Prospero in maintaining cell cycle arrest in the socket and the sheath cells. After CycE overexpresssion, (1) supplementary cell divisions were observed only in those terminal cells in which the Notch pathway is not endogenously activated, (2) supplementary cell divisions were observed in sheath cells and neurons in a *pros^17/+^* background; (3) activation of the N pathway blocked the ectopic division of the shaft cell; (4) socket cells underwent an extra division after reduction in N pathway activity and (5) additional sheath cells underwent extra divisions when both N and Pros activity was reduced.

In this study, the proliferative capacity of terminal cells was analysed when cell divisions were forced by CycE overexpression under different Notch and Prospero backgrounds. The analysis was performed under conditions in which either no cell transformation *per se* was observed (as in *pros* null background or *pros* heterozygous pupae) or in which only a small proportion of cells were transformed (as after mild overexpression of Numb or N^intra^). As such, the observed effects were considered to be the result of N and Pros acting directly on the maintenance of the state of cell cycle arrest rather than a modulation of cell fate acquisition. Furthermore, we never observed changes in bristle cell identities after CycE overexpression. This is in contrast to *Drosophila* neuroblasts, in which CycE seems to control cell identity independently of its role in the cell cycle [29]. Thus, in the bristle lineage system, CycE seems to act exclusively as a cell cycle regulator.

When N-activity was impaired and cell divisions were forced, both pIIa daughter cells divided like their mother, pIIa, each producing a shaft and a socket cell. These observations reveal that, just after birth, pIIa daughter cells are not yet committed to their final fate and retain pIIa characteristics. These results suggest that cell fate in pIIa daughter cells is acquired in a sequential manner. Initially, both pIIa daughter cells appear to be equivalent. Then, the N-pathway is rapidly activated in one daughter cell due to a N-signal originating from its sister cell [30]. The activation of the N-pathway arrests the cell cycle and this cell then acquires a socket terminal fate. Later on, the future shaft cell is committed, by a cell cycle independent and as yet unknown mechanism, to its terminal cell fate and becomes post-mitotic. Taken together, our results suggest that initially, precursor cell division is self-renewed and that an active process is required to commit both daughter cells to their final identities.

Our results show that 15 minutes after birth, pIIa daughter cells are committed to their normal fate and do not respond to CycE overexpression. This suggests that extra divisions observed in these cells result from a delay in the cell cycle exit rather than a re-entry into the cell cycle. In contrast, *cycE* expression induced extra mitosis even in fully differentiated neurons that were identified by 22C10/Futsch immunodetection [15]. Interestingly, these extra mitoses were always symmetric and gave rise exclusively to neurons. Similarly, extra divisions of sheath cells, under conditions that impaired the activity of Pros and N, were mainly symmetric producing two sheath cells. This suggests that sheath cells, like neurons, can also re-enter the cell cycle long after being committed to their final fate. Similar mitotic capacity has been also observed in other differentiated cells, in particular in neurons, suggesting that terminal differentiation and cell cycle exit are distinct events [31]–[35]. Why pIIIb daughter cells only divide symmetrically and how this characteristic is related to the capability of these cells to retain proliferative capacities are open questions.

The fact that CycE induced extra divisions in terminal cells suggests that these cells are arrested in G1. Two main mechanisms trigger G1-arrest: repression of Cyclin/Cdk2 activity by CKI and repression of E2F activity by RB proteins (reviewed in [36]). Both of these mechanisms are involved in the maintenance of a quiescent state. Our data show that in both neurons and future shaft cells of the sensory organs, a high level of CycE alone is sufficient to induce extra mitoses. This is in contrast to differentiated neurons of the eye and the anterior wing margin where extra mitoses after sustained *cycE* expression were induced only in a *rbf1* mutant background [6]. We suggest that, in our conditions CycE overexpression override the action of downregulateurs like Rbf1. Alternatively, factors other than Rbf1 can regulate cell cycle exit and the quiescent state maintenance in neurons and sheath cells. Interestingly, sensory organs containing two neurons were observed in null *dap* background (AA unpublished results). Thus, Dap is involved in neuronal cell arrest. Since Dap is also expressed in shaft cells [20], this factor probably plays a similar role in these cells.

Our data show that Prospero, together with N, cooperate in maintaining a quiescent state in sheath cells. Without overexpression of CycE, ectopic cell divisions occurred resulting in supplementary pIIIb daughter cells when Pros and N activity was reduced. Since the future neuron is a N-off cell, we expected that this cell will not be affected by the reduction in the N-function. As such, we anticipate that the future sheath cell undergoes a supplementary division. Thus, clusters containing two sheath cells and those containing two neurons would result from symmetric and asymmetric cell divisions respectively. Since these data were obtained in a non-sensitized condition, these results suggest that Pros and Notch are actively involved in maintaining a quiescent state in terminal cells during the normal development of bristles.

Between Pros and Notch, the former appears to predominate over the later to restrict cell proliferation. Indeed, in pIIIb daughter cells, CycE overexpression induced few extra divisions when N activity was reduced and significantly increased extra divisions in *pros* heterozygous cells. A role for Pros in maintaining cell cycle arrest is in agreement with the observation that neuronal proliferation was increased in *pros* loss of function embryos. It has been shown that this proliferation was associated with both an upregulation of *cycE*, *stg* and *e2f* genes [25] and a delay in the appearance of *dap* transcripts [26]. In our system, cell cycles were not resumed in *pros* null clusters *per se*. In addition, we observed only a mild increase in CycE expression and no decrease in Dap expression inside *pros* null clones. It is likely that in the absence of Dap downregulation, the increase in CycE levels is insufficient to induce extra mitosis.

N-signalling is pleiotropic and either promotes or represses cell cycle progression depending on the cellular context [37]. Thus, in *Drosophila* eye development, N-activity is necessary and sufficient to trigger cell cycle progression in G1 arrest in cells of the morphogenetic furrow, by derepressing the inhibition of E2F1 by RBF1 [38],[39]. Furthermore, glial precursor cells are maintained in an undifferentiated proliferative state by both N pathway activation and Dap downregulation [40]. In contrast, in follicle and wing cells, the N pathway has an anti-mitotic action [24],[41]. In follicle cells, N activity stops mitotic cycles and promotes endocycles by repressing the expression of both String/cdc25 and Dap and upregulating Fizzy-related/Cdh1 expression [23],[24]. In wing disc cells, N promotes G1-arrest by reducing E2F activity [41],[42]. The mechanism involved in this G1-cell cycle arrest seems to involve downregulation of the *dmyc* proto-oncogene and the *bantam* micro-RNA, both of which act positively on E2F activity [42]. If a similar mechanism is involved in the N-mediated G1-arrest observed in socket and sheath cells, we anticipate that *dmyc* and *bantam* would be downregulated exclusively in N-on bristle cells, in particular in pIIa, sheath and socket cells. However, the analysis of *dmyc* and *bantam* expression in sensory organ cells was not consistent with this idea. Unexpectedly, bantam and dMyc were detected in N-on cell such as pIIa, socket or sheath cells (AA, not shown). Thus, the mechanisms by which N and Prospero maintain cell cycle arrest in terminal cells of the bristle organ remain to be elucidated.

In conclusion, our results demonstrate that fate determination factors such as Notch and Prospero participate in maintaining a quiescent state in terminal cells. Under normal conditions, several mechanisms act in concert to ensure cell cycle arrest. Dap appears as a plausible candidate to ensure this role in N-off cells like the neuron and the shaft cell. N cooperates to maintain a quiescent state in sheath and socket cells and, finally, Pros acts only on the sheath cell. Consequently, the terminal quiescent state and cell differentiation do not seem to be regulated by mutually exclusive mechanisms. We favour the notion that these phenomena are regulated by parallel mechanisms involving factors having dual actions on fate acquisition and cell cycle progression.

## Materials and Methods

### Fly strains

The *neur*-GAL4 driver was used to specifically express H2B::YFP [14]; Partner Of Numb::GFP (PON::GFP, [17]), and CycE were expressed using the UAS/Gal4 system [13]. CycE overexpression was carried out using a line harbouring two copies of the UAS-*CycE* construct, one on the second and one on the third chromosome [5]. To increase viability of the *neur>CycE* and *neur>CycE*, *pros^17/+^* pupae, overexpression was performed using a strain bearing GAL80^ts^ (gift from D. Coen). Fly crosses, embryonic and larval development were carried at 18°C, and white pupae were transferred to 30°C to allow the expression of GAL4. Overexpression induced by heat-shocks were performed using the hs-*CycE* (Bloomington); hs-*numb* [21]; hs-*Nintra* [22] lines. In order to precisely stage pupae, pupae were collected at puparium formation and timed while considering that the developmental time at 18°C was twice longer than that at 25 or 30°C. Heat shocks were performed at 34°C or 38°C for 30 min and pupae were kept at 25°C for recovery. Somatic clones were obtained using the FLP/FRT recombination system [43]. The FRT82B *pros^17^* [27] line alone or combined with *N^ts-1^* [28] was crossed to the *y*, *w*,*Ubx*FLP; FRT82B ubi-nls::GFP (gift of J. Knoblich) to generate *pros*-null somatic clones.

### Time lapse in vivo imaging

Live imaging of the bristle lineage in *neur>CycE*, UAS-H2B::YFP, UAS-PON::GFP pupae was carried out as described previously [11]. Images were acquired every 3 minutes on a confocal microscope (20× or 40× objective) driven by Metaview (Universal Imaging). Temperature was maintained at 25°C. Time-lapse movies were assembled using ImageJ (free software). Sensory clusters were identified according to their relative positions on the thorax. For each mitosis, asymmetric localisation of the PON::GFP fusion protein allowed the identification of the daughter cells. Live imaging in experiments combining time-lapse imaging and immunodetection was realised using an spinning disc microscope (Ropert Scientific France) (20× or 40× objective) driven by Metaview (Universal Imaging). Images were acquired every 4 minutes. Temperature was controlled by a thermo-regulated chamber (home-made).

### Immunohistology

Pupal nota were dissected between 17 h and 35 h APF and processed as previously described [9]. Primary antibodies were: mouse anti-Cut (DSHB, 1∶500); rat anti-CycE (gift from H. Richardson, 1/1000); rabbit anti-Dap (gift from C. Lenher, 1∶300); rat anti-ELAV (DSHB, 1∶10); mouse anti-ELAV (DSHB, 1∶100); mouse anti-Futsch (22C10) (DSHB, 1∶100); rabbit anti-GFP (Interchim, 1∶1000); mouse anti-GFP (Roche, 1∶500); mouse anti-Pros (gift from C. Doe 1∶5); rat anti-Su(H) (gift from F. Schweisguth, 1∶500); rabbit anti-phospho-Histone H3 (Upstate, 1∶10000). Alexa 488- and 568-conjugated secondary anti-mouse, anti-rat, anti-rabbit, anti-guinea pig were purchased from Molecular Probe and used at 1∶1000. Cy5 conjugated antibodies anti-mouse, -rat or -rabbit were purchased from Promega and were used at 1∶2000. In addition to antibody immunodetection, we also used other criteria to identify cells. (1) Nuclear size, bigger in pIIa daughter cells than in pIIb daughter cells. (2) Cell location relative to both the antero-posterior axis and the cell arrangement into the cluster, socket cell posterior to shaft cell and both cells posterior to pIIb daughter cells. (3) Relative YFP intensity, neuron nucleus is less intense than that of the sheath nucleus, (4) Small and bright YFP staining, reflecting apoptotic DNA condensation, to distinguish the glial cell. Images were processed with ImageJ and Photoshop. Counting of terminal cells was carried on-fixed pupae and was restricted to the clusters forming the two middle rows (24 and 28 h APF or after one night recovery when a heat shock was applied).

## Supporting Information

Figure S1 CycE expression driven by *neur-Gal4*. Immunodetection of CycE in *neur>CycE* pupae at 23 h (A), 32 h (B) and 48 h APF (C) and at 23 h APF in (D) *hs-numb*, *neur>CycE*, (E) *hs-Nintra*, *neur>CycE* and (F) *pros^17/+^*, *neur>CycE* pupae. Sensory cells are revealed by GFP (green, UAS-H2B::YFP in all cases) and sheath cells by Prospero (blue) immunoreactivity. CycE immunoreactivity is in red and in black (inverse colour) in A'–F'. (D,F) Pupae were heat shocked at 20 h APF and dissected three hours later. Note that CycE accumulation fades away in the neurons at 48 h APF (arrowheads in C).
(8.94 MB TIF)

Figure S2 Time-lapse observation of a *neur>CycE*, *H2B::YFP* pupa. (A) Representative frames from a time-lapse observation of a *neur>H2B::YFP; Pon::GFP* pupae (control). (B) Representative frames from a time-lapse observation of a *neur>CycE; H2B::YFP; PON::GFP* pupae. Arrows indicate extra divisions. Abbreviations: g, glial cell; n, neuron; s, sheath cell; so, socket cell; sh, shaft cell. Time (h/min) APF is indicated in each panel. Anterior is upwards and the view is dorsal.
(3.91 MB TIF)

Figure S3 *pros^17^* clonal analysis. (A–C) Clones were detected by the lack of GFP staining (green), their limits are shown with a white dotted line. Sensory organs are identified with anti-Cut (red, A; blue, B, C) antibodies. (A) Four cells are present in all sensory organs (red) in *pros^17^* somatic clones. Nota from pupae at 28 hr APF. (B) CycE immunoreactivity (red) was more intense in sensory organs inside the *pros^17^* clone (arrows) than outside the clone (arrowhead). Nota from pupae at 22 hr APF. (C) Dap expression (red) was unchanged in sensory organs in- or outside of *pros^17^* clones. Arrowheads show the pIIIb cells. Nota from pupae at 21 hrs APF.
(7.67 MB TIF)

Video S1 *In vivo* observation of a *neur>H2B::YFP*, *PON::GFP* pupa (control). H2B-YFP is in green, PON-GFP is in red. Anterior is upwards and the view is dorsal.
(4.66 MB MOV)

Video S2 *In vivo* observation of a *neur>CycE*, *H2B::YFP*, *PON::GFP* pupa. H2B-YFP is in green, PON-GFP is in red. Arrows indicate extra divisions. Anterior is upwards and the view is dorsal.
(35.59 MB MOV)

Video S3 *In vivo* observation of a *neur>CycE*, *H2B::YFP*; *pros^17+/−^* pupa. Arrows indicate extra divisions and asterisk the glial cell death. The view is dorsal.
(8.90 MB MOV)
