# Supplementary figures and images for: Notch and Prospero Repress Proliferation following Cyclin E Overexpression in the Drosophila Bristle Lineage
Source: PLoS Genet. 2009 Aug 7;5(8):e1000594. doi: 10.1371/journal.pgen.1000594 (PMC2715135; doi:10.1371/journal.pgen.1000594)

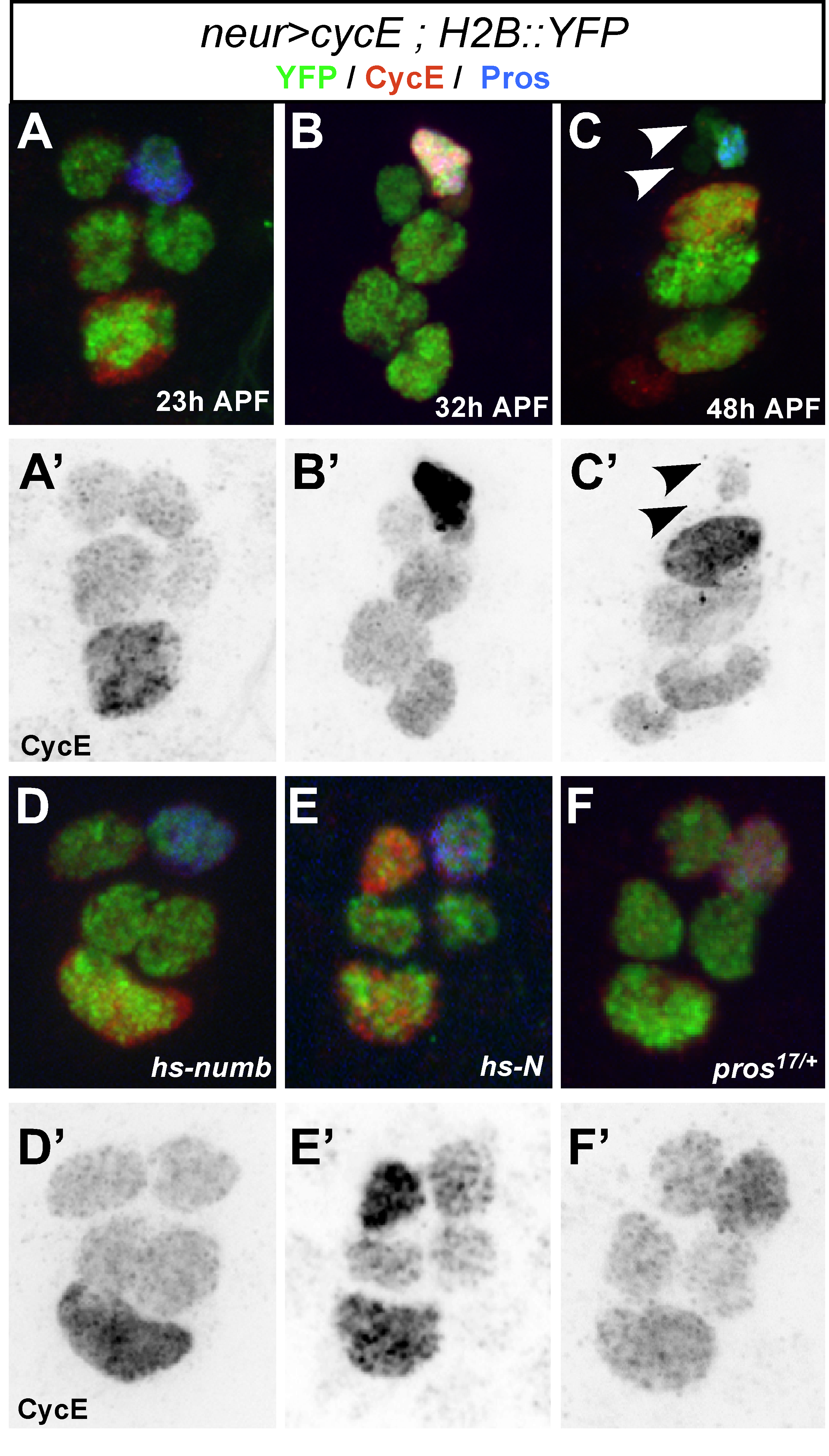

Supplement: Figure S1 — CycE expression driven by neur-Gal4. Immunodetection of CycE in neur>CycE pupae at 23 h (A), 32 h (B) and 48 h APF (C) and at 23 h APF in (D) hs-numb, neur>CycE, (E) hs-Nintra, neur>CycE and (F) pros17/+, neur>CycE pupae. Sensory cells are revealed by GFP (green, UAS-H2B::YFP in all cases) and sheath cells by Prospero (blue) immunoreactivity. CycE immunoreactivity is in red and in black (inverse colour) in A'–F'. (D,F) Pupae were heat shocked at 20 h APF and dissected three hours later. Note that CycE accumulation fades away in the neurons at 48 h APF (arrowheads in C). (8.94 MB TIF) [file pgen.1000594.s001.tif]

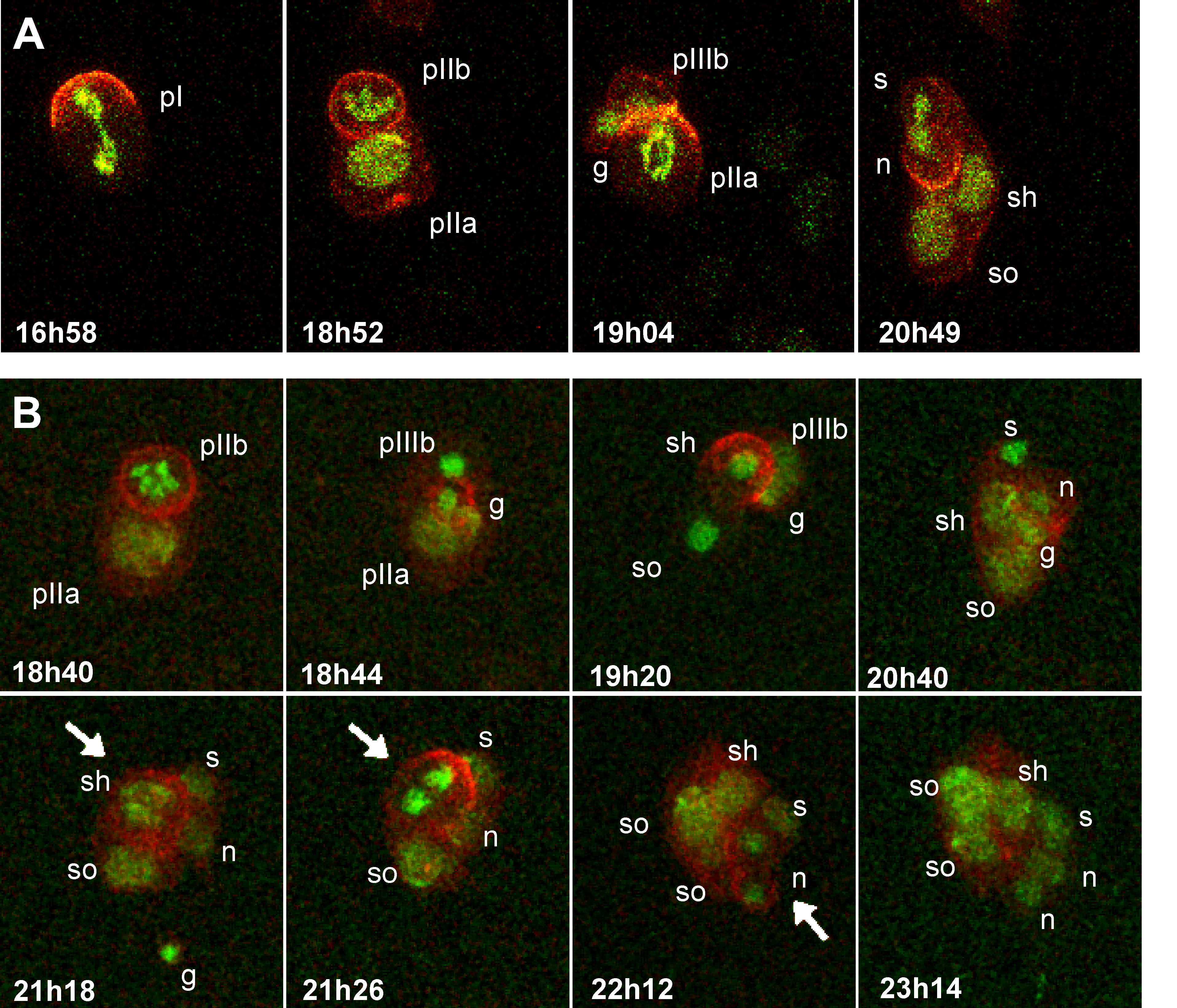

Supplement: Figure S2 — Time-lapse observation of a neur>CycE, H2B::YFP pupa. (A) Representative frames from a time-lapse observation of a neur>H2B::YFP; Pon::GFP pupae (control). (B) Representative frames from a time-lapse observation of a neur>CycE; H2B::YFP; PON::GFP pupae. Arrows indicate extra divisions. Abbreviations: g, glial cell; n, neuron; s, sheath cell; so, socket cell; sh, shaft cell. Time (h/min) APF is indicated in each panel. Anterior is upwards and the view is dorsal. (3.91 MB TIF) [file pgen.1000594.s002.tif]

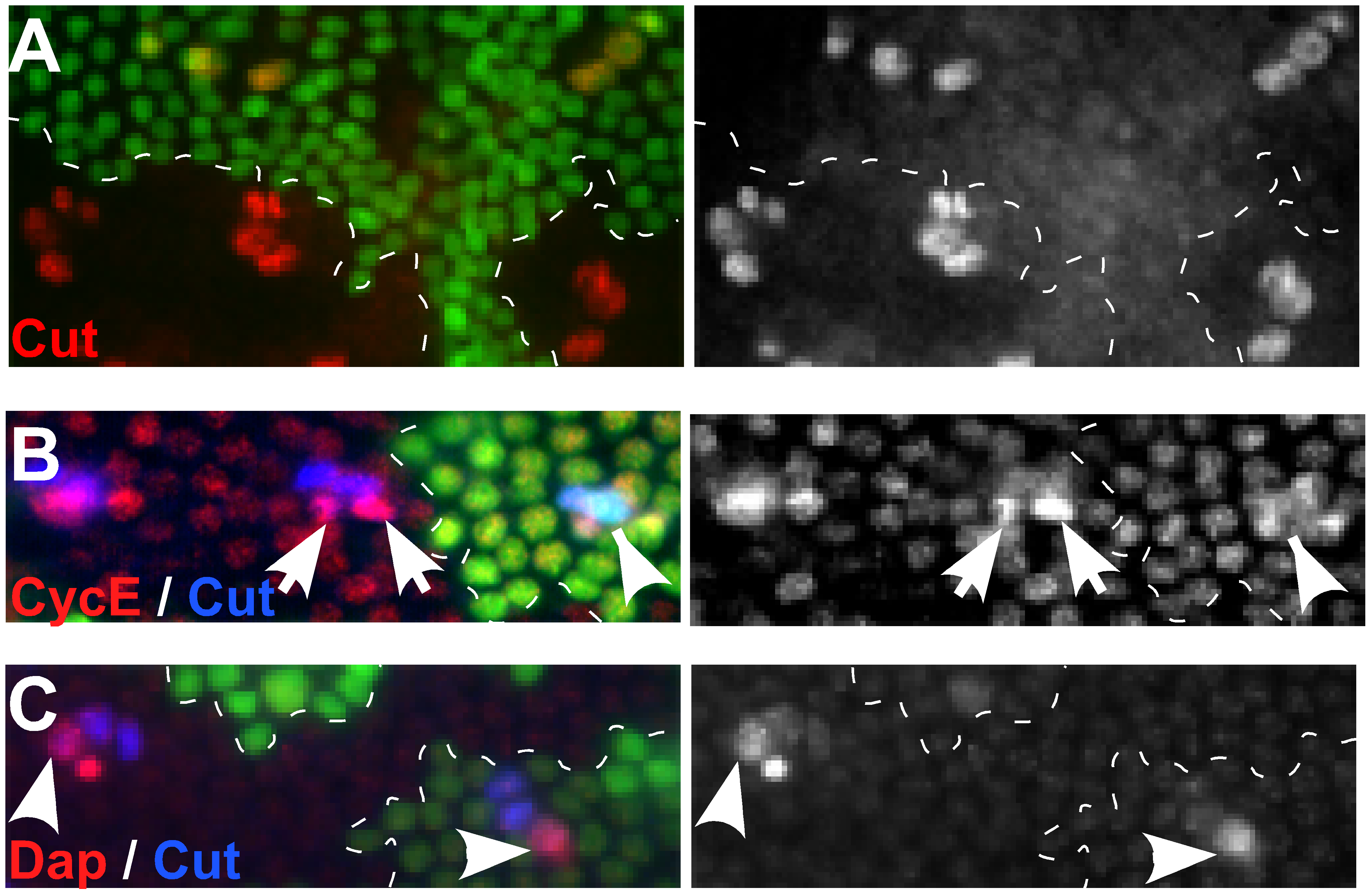

Supplement: Figure S3 — pros17 clonal analysis. (A–C) Clones were detected by the lack of GFP staining (green), their limits are shown with a white dotted line. Sensory organs are identified with anti-Cut (red, A; blue, B, C) antibodies. (A) Four cells are present in all sensory organs (red) in pros17 somatic clones. Nota from pupae at 28 hr APF. (B) CycE immunoreactivity (red) was more intense in sensory organs inside the pros17 clone (arrows) than outside the clone (arrowhead). Nota from pupae at 22 hr APF. (C) Dap expression (red) was unchanged in sensory organs in- or outside of pros17 clones. Arrowheads show the pIIIb cells. Nota from pupae at 21 hrs APF. (7.67 MB TIF) [file pgen.1000594.s003.tif]
